# *Coxiella burnetii* and Reproductive Disorders in Cattle: A Systematic Review

**DOI:** 10.3390/ani14091313

**Published:** 2024-04-27

**Authors:** Philippe Gisbert, Irina Garcia-Ispierto, Luis Angel Quintela, Raphaël Guatteo

**Affiliations:** 1 Ceva Santé Animale, 10, Avenue de la Ballastière, CS30126, 33500 Libourne, France; 2 Veterinària-Ciència i Producció Animal, Campus ETSEAFIV, Universitat de Lleida, 25198 Lleida, Spain; irina.garcia@udl.cat; 3 Reproduction and Obstetrics, Department of Animal Pathology, Faculty of Veterinary Medicine, Universidade de Santiago de Compostela, 27002 Lugo, Spain; luisangel.quintela@usc.es; 4 IBADER, Universidade de Santiago de Compostela, 27002 Lugo, Spain; 5 Oniris, INRAE, BIOEPAR, 44300 Nantes, France; raphael.guatteo@oniris-nantes.fr

**Keywords:** Q fever, *Coxiella burnetii*, cattle, reproduction, abortion, metritis, retained foetal membranes, fertility

## Abstract

**Simple Summary:**

Q fever is an infectious disease in ruminants caused by the bacterium *Coxiella burnetii*, which can be transmitted to humans. A review of the scientific literature was carried out to examine the relationship between Q fever and reproductive problems in cattle [abortion/stillbirth/perinatal morality/weak calves; non-expulsion of placenta; uterine infection; infertility/sub-fertility]. For each problem, a scientific evidence score was calculated for each eligible study to help reach a conclusion on the level of evidence for the impact of *C. burnetii*, if any, on the reproductive problem assessed. The literature search yielded 443 results, but only 61 were deemed eligible for use. There were 43 studies that looked at Q fever and abortion/stillbirth/perinatal morality/weak calves. For non-expulsion of the placenta, uterine infection and infertility/sub-fertility, there were 9, 8 and 19 studies, respectively. For abortion/stillbirth/perinatal morality/weak calves, non-expulsion of placenta and infertility/sub-fertility, there is a good deal of evidence to support the involvement of Q fever. In contrast, for uterine infections, the evidence is unclear. There is a strong need for more research, particularly involving larger numbers of study animals. To provide more consistency in this field of study, it is recommended that scientists follow more precise definitions of reproductive problems and use better methodologies to test for Q fever infection.

**Abstract:**

Coxiellosis or Q fever is an infectious zoonotic disease caused by the bacterium *Coxiella burnetii*. A systematic review using bibliographic research was carried out, and the focus was the relationship between *C. burnetii* infection and reproductive disorders in cattle [abortion/stillbirth/perinatal morality/weak calves (ASPW complex); retained foetal membranes (RFMs); metritis/endometritis; and infertility/sub-fertility]. The bibliographical search yielded 443 results from databases, but only 61 were deemed eligible. For each disorder, summary tables were prepared, and a scientific evidence score was calculated for each study based on four criteria to help assess the level of evidence for the impact of *C. burnetii* on the reproductive disorders assessed: type of publication (peer-reviewed or other); type of study (case–control/cohort or other); type of *C. burnetii* test (direct or indirect); and comparative statistical analysis (yes or no). In addition, summary tables also included information on the study population, country, authors and year of publication, key findings and an assessment of the evidence for an association. For the ASPW complex, RFMs, metritis/endometritis and infertility/sub-fertility, 43, 9, 8 and 19 studies provided data, respectively. On a scale of four, nearly 50% of all study citations had evidence scores of three or four. For ASPW, RFMs and infertility/sub-fertility, there is a significant body of evidence to support a deleterious role for Q fever. In contrast, for metritis/endometritis, the evidence is unclear. It is concluded that there is a substantial need for further research, particularly involving larger animal populations in more controlled settings. To provide more consistency, it is recommended that authors follow more precise definitions of reproductive parameters and more robust diagnostic methodologies.

## 1. Introduction

Coxiellosis or Q fever is an infectious zoonotic disease caused by the bacterium *Coxiella burnetii* [1]. Human infections are often asymptomatic, but they can sometimes cause serious clinical signs, which can include headaches, flu-like illness, chills, pneumonia, fatigue syndrome, weight loss, nausea and muscle soreness. In some cases, a heart infection (pericarditis, myocarditis or endocarditis) or hepatitis can also appear. In some other rare cases, persistent focalized infections can occur and these include endocarditis, vascular infection, prosthetic joint arthritis, osteoarticular infection (without prosthesis) and lymphadenitis [2].

The European Food Safety Authority (EFSA) proposed the following Q fever case definition in animals: the existence of various reproductive problems, including abortion and perinatal mortality; confirmation of the presence of *C. burnetii* in vaginal/placental/aborted tissue swabs from aborted females using PCR; and seropositivity in several other females within the herd [3]. However, this definition should be treated with caution since serology lacks sensitivity [4], and a previously published review highlighted the involvement of *C. burnetii* in other reproductive disorders [5]. Furthermore, the latest World Organisation for Animal Health (WOAH) Terrestrial Manual stipulates that “*C. burnetii* might be associated with metritis and infertility in cattle” [6]. The manual describes the pathogen’s persistence, often lifelong, and its tendency to cause sporadic or epidemic abortions and the birth of dead or weak offspring, with recovery typically uncomplicated.

In small ruminants, the dominant effect on reproduction is abortion and stillbirth [7,8]. However, in cattle, some other reproductive disorders have also been described, like retained foetal membranes (RFMs), metritis/endometritis and infertility/sub-fertility [5,9]. These additional signs have not been studied thoroughly in small ruminants. The impact of Q fever on reproduction in cattle has been investigated in the scientific literature. *C. burnetii* demonstrates a tendency for shedding through diverse routes, including milk, faeces, parturition products (comprising placental tissue and aborted foetuses) and vaginal mucous. This highlights the complex interaction between Q fever and bovine reproductive processes. Consequently, this systematic review aims to synthesize existing knowledge on the pathological impact of Q fever on abortion, stillbirth, perinatal mortality and weak offspring (collectively referred to as ‘ASPW complex’), RFMs, metritis/endometritis and infertility/sub-fertility, thereby contributing to the understanding of the dynamics governing the intersection of Q fever and bovine reproductive health. Although previous reviews [5,9] have been published about the association between *C. burnetii* and reproductive disorders in cattle and other domestic animals, many related cattle studies have been published over the last 10 years; thus, it is considered that a new review would be timely and of interest. Early diagnosis is crucial to prevent health issues in animals. Beyond abortion, it is essential to identify signs attributable to *C. burnetii*, differentiating bacterial shedding from disease manifestation, to develop an evidence-based understanding of Q fever’s impact on cattle reproduction.

Specifically, the aim of this review was to systematically review the existing literature on the clinical manifestations of *C. burnetii* in reproduction in cattle. Specifically, this included an assessment of the relationships between *C. burnetii* infection and the ASPW complex, RFMs, metritis, endometritis, infertility and sub-fertility.

## 2. Materials and Methods

### 2.1. Definition of the Reproductive Parameters

Abortion is generally defined as the interruption of a pregnancy between day 42 and 260 of gestation [10,11]. Pregnancy losses not only seriously affect the cattle industry’s ability to reproduce effectively but also indicate poor cattle welfare and possible health issues due to various zoonotic agents [12,13,14]. In contrast, stillbirth in calves is defined as the death of a foetus shortly before or during calving at full term (≥260 days). Perinatal mortality is usually defined as the death of the foetus or calf before, during or within 48 h of calving at full term and may include stillbirths in some studies [15]. Finally, weak offspring would be considered as calves born alive but with a lack of vigour (e.g., cannot rise to suckle or die within a few hours of birth). Placental retention is the failure to expel foetal membranes within an acceptable time after parturition. Depending on the authors and studies, the length of this period can vary from 12 to 48 h [16,17]. Puerperal metritis and endometritis have previously been defined [18]. Briefly, puerperal metritis (also called metritis) is a uterine disease characterized by a watery and fetid discharge associated with systemic clinical signs, including fever above 39.5 °C within 21 day after parturition. In contrast, clinical endometritis appears later (after 21 d) and is characterized by a purulent or muco-purulent discharge into the vagina. No systemic and local clinical signs are associated with sub-clinical endometritis (i.e., no discharge but leucocytes present on endometrial swabs). In strict terms, infertility is the inability to produce viable offspring. If this inability is permanent, we would refer to it as sterility, and if we are talking about difficulty rather than incapacity, we would be referring to sub-fertility. In the case of farm animals, infertility is defined as the inability to produce viable offspring within a defined period, which would impact the economic profitability of the animal. For example, in the case of cows, it is ideally considered appropriate for them to have one calf per year. In this case, infertility and sub-fertility would essentially be equivalent. To have one calf per year, it is necessary to inseminate cows for the first time after calving at 60 day and for them to become pregnant by day 85, with a maximum of 1.8 inseminations per gestation [19,20]. Based on this, there are indicators that must be met for cattle farms to be considered free of an infertility problem. For example, calving to first service interval, first or all services pregnancy rates or calving to conception interval [21]. The appropriate values for these indicators will depend on different factors, including the breed, production level or management system of the farm, so they may vary among farms. 

Furthermore, they can in some cases be the consequence of other conditions, such as metritis or endometritis, or the number of repeat breeders and calving-to-calving interval [19,20]. The EFSA definition mentioned in the introduction is very precise but there are lots of other definitions in the literature. Similarly, there are many quite precise definitions of the reproductive parameters examined in this review. However, given that the number of publications available is limited, no publications were excluded because of differing definitions of the reproductive parameters. Therefore, the parameter definitions were accepted as they are defined by the authors of the studies. 

### 2.2. Systematic Review

For this systematic review, the bibliographic search was carried out according to PRISMA guidelines [22]. It was decided to focus the search on the relationship between *C. burnetii* infection and reproductive disorders, including the ASPW complex, RFMs, metritis/endometritis and other reproductive disorders (infertility/sub-fertility). It was voluntarily decided not to include udder health or respiratory disease, although these two conditions have also been described in the literature [23,24].

Using the PICO (population, intervention, comparison, outcomes) model [25], the specific question addressed by the review is as follows: “Is *C. burnetii* responsible for various reproductive disorders in cattle?”

- Population: cattle, specifically dairy cows and breeding heifers.
- Intervention: reproductive disorders (ASPW complex, RFMs, metritis/endometritis, infertility/sub-fertility).
- Comparison: none.
- Outcomes: relationship between clinical signs and *C. burnetii* infection/prevalence or/and mechanism of action.

The Dialog tool (Proquest, Ann Arbor, MI, USA) was used, and five databases (BIOSIS Previews, British Library Inside Conferences, MEDLINE, CAB Abstracts and Publicly Available Content) were included. We searched for articles containing in their title or abstract all terms relating to *C. burnetii*/Q fever/coxiellosis, a term relating to reproductive disorders or clinical signs in relation to reproduction and a term referring to cattle. Thus, we defined the following query: (ab,ti(q fever) OR ab,ti(*Coxiella*) OR ab,ti(burneti*) OR ab,ti(coxiellosis)) AND (ab,ti(abortion) OR ab,ti(stillbirth) OR ab,ti(weak*) OR ab,ti(premature) OR ab,ti(*fertility) OR ab,ti(*metritis*) OR ab,ti(foetal-membranes) OR ab,ti(placenta*) OR ab,ti(pregnancy)) AND (ab,ti(cattle) OR ab,ti(cow*) OR ab,ti(heifer*) OR ab,ti(bovin*)). The search was run in “command line” mode on the 16 October 2023 with the duplicate removal option enabled. Abstracts from the last two World Buiatrics Congresses (WBC 2018 and WBC 2022) have also been included. Note that the previous congresses were not included as we considered that older relevant data should have been published by now in a peer-reviewed journal after their presentations at the congress. The proceedings of these two recent congresses were reviewed. The search function of Acrobat Reader (Adobe, San José, CA, USA) was used. All the abstracts containing at least one of the following expressions were retained: “q fever”, “Coxiella” and “coxiellosis”. 

Specifically, the articles included had to (a) present primary data (e.g., no review papers), (b) include data on the diagnosis of Q fever, (c) present data collected from cattle and (d) include data on one or more of the clinical signs included in the review scope. Articles not written in English or French were translated with the help of the https://translate.google.com (accessed on 23 October 2023) application (Google Ireland Limited, Dublin, Ireland) or https://www.deepl.com/fr/translator (accessed on 23 October 2023) (DeepL SE, Cologne, Germany). A first selection was made from the abstracts with all abstracts checked to confirm which met all inclusion criteria. In the second step, the full texts of these selected articles were read by the first author. If, despite the search criteria, the abstract or the full text did not meet our criteria, it was rejected. For example, an article relating to human Q fever causing abortion in pregnant women and mentioning that cattle could be the source of this contamination could appear in the bibliographic search but was beyond the scope of our review. 

In the following study summary tables, a scientific evidence score (up to 4: ****) has been calculated by the authors for each study based on four major criteria to help reach a conclusion on the level of evidence for the impact of *C. burnetii*, if any, on the reproductive disorders assessed:

- Type of publication: peer-reviewed (PR *); other publication (OP ^).
- Type of study: case–control and cohort studies (CCS *); other type of study (without a control group; OTS ^).
- Type of *C. burnetii* test: direct test (PCR, histology, ELISA antigen) on individual samples (DIR *); indirect test (antibodies based on serology) or direct test on a sample of pooled animals (including Bulk Tank Milk—BTM) (OTH ^).
- Comparative statistical analysis: Yes *; No ^.

If all four criteria are met (PR, CCS, DIR and Yes), a score of **** is assigned; if one criterion is not met: ***^; if two are not met: **^^; if three are not met: *^^^; if no criteria are met: the total evidence score cell shows ^^^^. All evidence scores contain four characters, either * or ^ and the characters are placed in the order of the assessed parameters within the table columns. Because evidence scores were not assigned to the few studies specifically related to the possible mechanism of action of *C. burnetii* on the ASPW complex, these studies do not appear in Table 1, Table 2 and Table 3.

In addition to the above four criteria, the summary results tables also include some information on the study populations, including numbers of animals and herds involved, the country/countries where the studies were performed, the authors and year of publication, the key findings and an assessment of whether the evidence is positive, negative or unclear. Although one could argue that the review criteria could have excluded all studies that did not have comparative statistical analyses, given the very limited number of articles available, it was decided to include but identify those articles and let the reader decide on their importance to our conclusions. Importantly, only one article (ASPW complex) [26] citation scoring three or four in the summary tables had no comparative statistical analysis.

## 3. Results and Discussion

### 3.1. Search Results

The bibliographical search yielded 443 results from databases. Twenty duplicates, in addition to those automatically removed by the Dialog tool, were manually removed. After the screening, 40 articles were found to be review articles (including 39 reviews and one bibliographic thesis), one article was a bibliometric analysis and two articles were letters to the editor. We also eliminated 303 other articles for different reasons: not being related to Q fever (*n* = 30), cattle (*n* = 33) or clinical signs (*n* = 18); being related to seroprevalence (*n* = 127), human medicine (*n* = 24), bacteriology or molecular biology (*n* = 17), control measures and surveillance programs of the disease (*n* = 18), laboratory techniques (*n* = 35) or mathematical modelling (*n* = 1). Finally, 83 articles were retained for full paper reading and evaluation. We were unable to obtain two articles. After further reading, 20 articles were eliminated because they were not related to Q fever (*n* = 3) or clinical signs (*n* = 4); or they were related to epidemiology (*n* = 8), control measures (*n* = 1), laboratory techniques (*n* = 3) or a survey of farmers on their perception of the disease (*n* = 1). 

**Figure 1:**
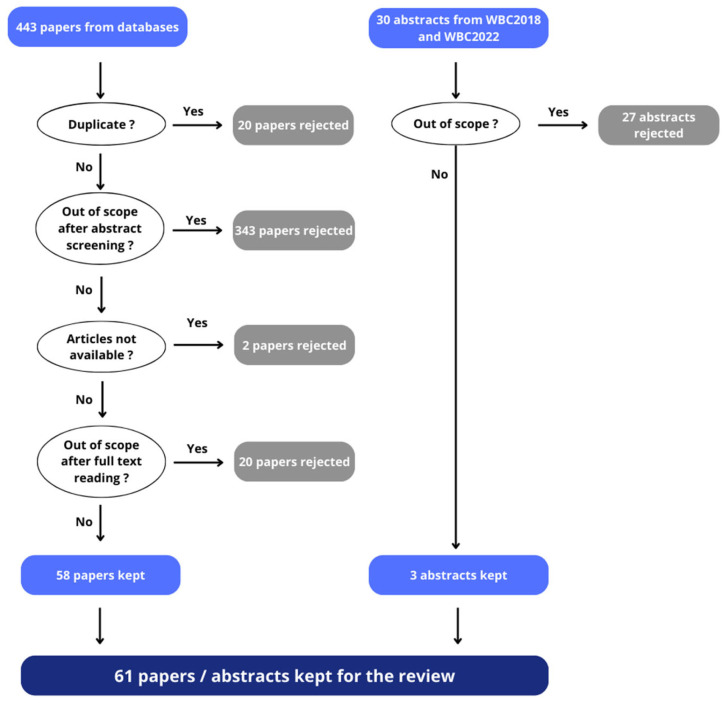
The article selection flow at each stage, showing the number of publications included and excluded at each level—from databases and from conference proceedings (WBC2018 and WBC2022).

Therefore, 58 papers from the comprehensive literature search of databases were retained. Three abstracts from conference proceedings were also included (Figure 1).

The literature search and review allowed us to assess the existing data concerning the impact of Q fever on reproduction in cattle. Table 1 summarizes the evidence scores from the various studies. Note that many studies examined several reproductive disorders and therefore appear in more than one disorder row. That is, there were 79 study citation evidence scores available from a total of 61 studies.

**Table 1:** Summary of the number of articles with their evidence score for each reproductive disorder examined (numbers with percentage of total).

Table	Parameter	Score 0	Score 1 (*)	Score 2 (**)	Score 3 (***)	Score 4 (****)	Total
Table 3	ASPW Complex	0 (0%)	4 (9%)	21 (49%)	15 (35%)	3 (7%)	43
Table 4	RFMs	0 (0%)	1 (11%)	5 (56%)	1 (11%)	2 (22%)	9
Table 5	(Endo)metritis	0 (0%)	0 (0%)	4 (50%)	3 (37%)	1 (13%)	8
Table 6	Infertility/Sub-fertility	0 (0%)	1 (5%)	6 (32%)	10 (53%)	2 (10%)	19

A scientific evidence score (0 to 4) was calculated for each study based on four major criteria to help reach a conclusion on the level of evidence for the impact of *C. burnetii*, if any, on the reproductive parameters assessed. For each criteria met (PR, CCS, DIR and Yes), a * is assigned.

Only 10% of all study citation evidence scores had scores of four (i.e., peer-reviewed publication, case–control study, direct test for *C. burnetii*, statistical analysis), with an additional 37% having a score of three. Table 2 shows the numbers and percentage of studies where there was a relationship between *C. burnetii* and a reproductive parameter.

**Table 2:** Summary of the incidence of studies that showed an association between *C. burnetii* and a reproductive parameter (numbers with percentage of total).

Table	Parameters	Studies with Evidence Scores of 1, 2, 3 or 4		Studies with Evidence Scores of 3 or 4	
		Evidence Available	No Evidence Available	Evidence Available	No Evidence Available
Table 3	ASPW Complex	33/43 (77%)	10/43 (23%)	13/18 (72%)	5/18 (28%)
Table 4	RFMs	5/9 (56%)	4/9 (44%)	2/3 (67%)	1/3 (33%)
Table 5	(Endo)metritis	3/8 (38%)	5/8 (62%)	2/4 (50%)	2/4 (50%)
Table 6	Fertility/Sub-fertility	12/19 (63%)	7/19 (37%)	8/12 (67%)	4/12 (33%)

A scientific evidence score (0 to 4) was calculated for each study based on four major criteria to help reach a conclusion on the level of evidence for the impact of *C. burnetii*, if any, on the reproductive parameters assessed.

### 3.2. Abortion, Stillbirth, Perinatal Mortality and Weak Offspring (ASPW Complex)

The available evidence for the association between *C. burnetii* and ASPW is outlined below. In addition, when available, based solely on the publications resulting from the search criteria, relevant data on possible mechanisms of action are mentioned. 

The first evidence of the involvement of *C. burnetii* in bovine abortion was provided by an experimental study in France [24]. In that study, 11 heifers were intra-dermally experimentally challenged with different doses of a suspension of *C. burnetii* (strain C9), and 53 heifers were in the control group. After insemination, 4 of the 11 challenged heifers had a normal gestation, resulting in a live calf. A total of 2 of the 11 heifers were slaughtered while pregnant, and the foetuses were normal. The remaining challenged heifers (*n* = 5; 45%) aborted, while, in the control group, 19% of the heifers aborted. The authors specified that no statistical analysis was performed because of the lack of rigour: some animals in the control group had not been inseminated. 

Most studies (77%) on cattle across multiple European, Asian, African and South American countries show a positive association between the presence of *C. burnetii* and the occurrence of the ASPW complex (Table 2 and Table 3), including those (72%) with evidence scores of three or four.

**Table 3:** Exhaustive list of studies (*n* = 43) related to the putative involvement of *C. burnetii* in the ASPW complex.

Country Authors Ref. No.	Type of Publication: PR or OP	Study Population: No. of Animals (A) and/or Herds (H)	Type of Study: CCS or OTS	Type of Test to Identify Presence of *C. burnetii*: DIR and/or OTH	Comparative Statistical Analysis: Yes or No	Key Findings	Evidence Score (^^^^ to ****)	Evidence for Association between ASPW and *C. burnetii:* Yes, No or Unclear
Algeria Derdour et al., 2017 [27]	PR *	A: 360; H: 54;	CCS *	OTH ^: Elisa on serum	Yes *	Seropositive cows are 7 times more at risk of abortion than seronegative cows (OR 7.08; CI 95% 1.27–39.36, *p* < 0.05).	**^*	Yes
Austria Sodoma et al., 2019 [28]	PR *	A: 150; H: 50–75	OTS ^	DIR * & OTH: ELISA on aborted cow serum (*n* = 85); PCR on aborted foetuses (*n* = 66)	No ^	Total of 5% of abortions in the study population was related to Q fever.	*^*^	Yes
Belgium Saegerman et al., 2015 [29]	PR *	H: 206	OTS ^	OTH ^: ELISA on Bulk Tank Milk (BTM)	Yes *	BTM seropositive herds (58%) are more likely to experience abortion (multivariate analysis; OR 2.04; 95% CI 1.14–3.66; *p* = 0.02) and stillborn or weak calves (univariate analysis; OR = 2.14; 95% CI: 1.05–4.39; no *p*-value) than seronegative herds.	*^^*	Yes
Bulgaria Vidic et al., 1990 [30]	PR *	A: 387 (134 had aborted)	OTS ^	OTH ^: Complement Fixation Test (CFT) on serum	Yes *	Seropositivity rate is higher in cows having had abortion (19.4%) than in randomly chosen cows (9.5%) (*p* < 0.01).	*^^*	Yes
Canada Bildfell et al., 2000 [31]	PR *	A: 43 (aborting cows)	CCS *	DIR *: Smear colouration and immunohistochemistry (IHC) of placentas	Yes *	*C. burnetii* positivity associated with inflammation (*p* = 0.003), necrosis (*p* = 0.012), and foetal pneumonia (*p* = 0.015).	****	Yes
Cyprus Cantas et al., 2011 [32]	PR *	A: 51 (aborting cows) H: 51	OTS ^	DIR *: PCR on aborted foetuses	Yes * (survey-data-analysis procedure)	Total of 35% of foetal stomach samples were PCR-positive. The logistic regression model identified ticks (OR = 4.5; *p* < 0.05), poor hygiene (OR = 0.30; *p* < 0.05) and presence of carnivores (OR = 3.3; *p* < 0.01) as the on-farm risk factors associated with occurrence of C. *burnetii* abortions.	*^**	Yes
Czech Republic Literak and Rodriguez 1994 [33]	PR *	A: 213 (aborting cows) H: 13	OTS ^	OTH ^: CFT on serum	Yes *	Total of 6% of aborted cows were seropositive; unclear of *C. burnetii* involvement.	*^^*	Unclear
Denmark Jensen et al., 2007 [26]	PR *	A: 86	CCS *	DIR *: Fluorescent in situ hybridization (FISH) and IHC of placentas	No ^	One placenta was positive.	***^	Unclear
Denmark Nielsen et al., 2011 [34]	PR *	A: 2362 calvings H: 24 with 13 BTM samples/H	CCS *	OTH ^: ELISA on BTM	Yes *	Total of 5.6% of calvings had perinatal mortality. Level of BTM positivity not associated with increased risk of stillbirth (>270 day of pregnancy) or perinatal mortality (<24 h after birth). Timing of BTM sampling may be important.	**^*	No
Ethiopia Robi et al., 2023 [35]	PR *	A: 461	CCS *	OTH ^: ELISA on serum	Yes *	Total 22.6% prevalence of abortion. Seropositive cows are more likely (OR 2.7; 95% CI 1.26–5.62; *p* < 0.05) to abort than seronegative cows.	**^*	Yes
France Durand and Strohl 1978 [36]	PR *	A: 2222 not aborted and 575 aborted cows	OTS ^	OTH ^: CFT on serum	No ^	Seropositivity rate ‘significantly’ higher in aborted cows than in non-aborted (4.1% vs. 1.8%; *p*-value not given).	*^^^	Yes
France Guatteo et al., 2012 [37]	PR *	A: 24 aborted cows	OTS ^	DIR * & OTH: PCR on vaginal mucous; ELISA on serum and milk	No ^	*C. burnetii* shedding in vaginal mucous of very short duration post-calving. Serum ELISA is a poor tool to diagnose abortions or detect shedder cows.	*^*^	Unclear
France Gache et al., 2017 [38]	PR *	A: 731 for serology, 2695 for abortion	OTS ^	DIR * & OTH: ELISA ± PCR on serum	No ^	Total of 2.7% (*n* = 90) of the abortive episodes investigated potentially related to *C. burnetii*.	*^*^	Yes
France Guatteo et al., 2020 [39]	PR *	H: 866 (series of abortions)	OTS ^	DIR * & OTH: PCR on aborted foetuses; ELISA on serum of six random cows within one herd	No ^	According to the French observatory for abortion causes in ruminants, *C. burnetii* is involved in 9.7% of cattle.	*^*^	Yes
France Jegou et al., 2022 [40]	OP ^	H: 642	OTS ^	DIR * & OTH: PCR on vaginal discharge; ELISA on serum	Yes *	Q fever involved in 12% of abortions.	^^**	Yes
Germany van Moll et al., 1993 [41]	PR *	A: 4	OTS ^	DIR *: IHC on foetal placenta	No ^	*C. burnetii* antigen found in placenta of aborted foetuses.	*^*^	Yes
Germany Sting et al., 2000 [42]	PR *	A: 826; H: 38	OTS ^	DIR * & OTH: Capture ELISA on vaginal swabs (antigen detection); CFT on serum	Yes *	No significant relationship between *C. burnetii* infection and abortion.	*^**	No
Germany Sting et al., 2002 [43]	PR *	A: 1167 (77 aborted); H: 105 (with fertility disorders)	CCS *	DIR * & OTH: Capture ELISA on vaginal swabs (antigen detection); ELISA on serum	Yes *	Infected cows (antigen detected) are more likely (OR 3.68; 95% CI 1.01–12.12, *p* = 0.01) to abort. Seropositive cows are more likely (OR 2.07; 95% CI 1.23–3.49, *p* = 0.003) to abort.	****	Yes
Germany Muller et al., 2015 [44]	PR *	A: 591; H: 48 (38 with abortion problems)	OTS ^	OTH ^: ELISA on serum	Yes *	The number of seropositive animals is higher in herds with abortion problems (*p* = 0.03).	*^^*	Yes
Germany Freick et al., 2018 [45]	PR *	A: 86 (56 having stillbirths); H: 1 (endemically infected)	CCS *	DIR * & OTH: PCR and ELISA on pre-colostral calf blood; PCR on cotyledons	Yes *	Total of 7.1% of stillbirth calves were PCR-positive; no live birth calves were positive. *C. burnetii* DNA found in 5/12 cotyledon samples of stillbirth cows vs. 2/12 live birth cows.	****	Yes
Greece Dovolou et al., 2011 [46]	PR *	H: 80 (for global seroprevalence) A: 526; H: 10 (for within-herd seroprevalence and relationship with fertility disorders	OTS ^	OTH ^: ELISA on BTM for global seroprevalence; ELISA on blood for individual seroprevalence	Yes *	Total of 35% (*n* = 28) of the farms have antibodies in BTM. No statistical relationship between animal serology status and abortion rate.	*^^*	No
India Balamurugan et al., 2021 [47]	PR *	A: 323; H: 44 (with history of abortion)	OTS ^	DIR * & OTH: PCR and ELISA on serum	Yes *	Overall seropositivity was 44%, of which 60% were associated with abortions (*p* < 0.01).	*^**	Yes
India Sarangi et al., 2021 [48]	PR *	A: 64; H: 1	OTS ^	DIR * & OTH: PCR on placenta, milk, vaginal swab, nasal swab; ELISA on serum	Yes *	*C. burnetii* DNA detected in 12/64 samples from abortion. Seropositivity and history of abortion were not associated (OR 0.94; 95% CI 0.4–2.2).	*^**	Unclear
Iran Mohabati-Mobarez et al., 2021 [49]	PR *	A: 46	OTS ^	DIR *: PCR on aborted foetuses (liver or spleen) or placenta	No ^	*C. burnetii* in 21.7% of aborted cattle samples.	*^*^	Yes
Italy Cabassi et al., 2006 [50]	PR *	A: 1250 (650 having aborted)	CCS *	OTH ^: ELISA on serum	Yes *	Seropositivity rate higher in aborting cows than in control cows (44.9% vs. 22%; *p* < 0.001).	**^*	Yes
Italy Valla et al., 2014 [51]	PR *	H: 246 (106 positive)	CCS *	OTH ^: PCR on BTM	Yes *	No relationship between positivity and the incidence of abortion (other causes of abortions were not investigated).	**^*	No
Italy Coin et al., 2022 [52]	OP ^	A: 4562 (all with aborted foetuses)	OTS ^	DIR * & OTH: PCR on foetal spleen; CFT and ELISA on serum	No ^	Total of 15% of cows had *C. burnetii* antibodies and it was detected in 4.7% of aborted foetuses.	^^*^	Yes
Latvia Grantina-Levina et al., 2022 [53]	PR *	A: 1557 (aborted cows) and 744 (foetuses) H: 1062	OTS ^	DIR * & OTH: PCR on aborted foetuses (liver, spleen, placenta; ELISA on serum and milk	Yes *	Seropositivity rate of aborted cows is significantly higher than for non-aborting cows (20.6% vs. 3.3%, *p* < 0.00001).	*^**	Yes
Netherlands Muskens et al., 2012 [54]	PR *	A: 100 (aborted/stillborn foetuses) H: 96	OTS ^	DIR *: PCR on pooled foetal tissues and placenta; IHC on placenta	No ^	Four placentas were IHC- and PCR-positive; all from third-trimester calves and associated with inflammation of the placenta.	*^*^	Yes
Pakistan Hussain et al., 2022 [55]	PR *	A: 448 (half cows, half buffaloes) H: 112	OTS ^	OTH ^: ELISA on serum	Yes *	Total 58.9% positivity of herds. There was an association between seropositivity and history of abortion (OR 8.87; 95% CI 5.39–15.10; *p* < 0.001).	*^^*	Yes
Portugal Clemente et al., 2009 [56]	PR *	A: 29 with history of abortion	OTS ^	DIR *: PCR on placenta (*n* = 1), vaginal swab (*n* = 4), foetal organs (*n* = 24)	No ^	Total of 17.2% of samples were seropositive.	*^*^	Yes
South Africa Mangena et al., 2023 [57]	PR *	A: 272	OTS ^	DIR * & OTH: PCR on foetal organs; CFT on serum	Yes *	Seropositivity of 0.7%. *C. burnetii* DNA found in organs of aborted foetuses and stillborn calves in 2/6 animal samples.	*^**	Yes
Spain Lopez-Gatius et al., 2012 [58]	PR *	A: 781 (>90 day in milk); H: 3 (history of sub-fertility and positive BTM tests)	OTS ^	OTH: PCR on BTM; ELISA on serum	Yes *	Seropositivity was detected in 50.2% of cows but was not associated with rates of abortion or stillbirth.	*^^*	No
Switzerland Hassig and Lubsen 1998 [59]	PR *	A: 6923 (with or without history of abortion); H: 352	CCS *	OTH ^: CFT on serum	Yes *	A total of 16.7% positive—no case/control difference. In infected herds, seropositive cows have a higher (OR 4.68; 95% CI 0.92–28.46) risk of abortion.	**^*	Yes
Switzerland Mock et al., 2020 [60]	PR *	A: 47; H: 21	OTS ^	DIR * & OTH: PCR on placenta and/or foetus abomasal contents; ELISA for antigen and antibodies on serum	Yes *	*C. burnetii* is a major cause of infectious perinatal mortality (found in 34.2% and 55.6% of primiparous and multiparous cows, respectively, more than any other of 13 pathogens tested for).	*^**	Yes
Turkey Kucukkalem et al., 2013 [61]	OP ^	A: 100	OTS ^ (Pilot study)	DIR *: PCR on aborted foetuses (spleen, liver or stomach contents)	No ^	*C. burnetii* DNA found in 6/100 liver and lungs of aborted foetuses.	^^*^	Yes
Turkey Gunaydin et al., 2015 [62]	PR *	A: 102	OTS ^	DIR *: PCR on aborted foetuses stomach contents	No ^	*C. burnetii* DNA in stomach of 3.9% of aborted foetuses.	*^*^	Yes
Turkey Yilmazbas-Mecitoglu et al., 2022 [63]	OP ^	A: 575 (174 positive, 226 negative)	CCS *	OTH ^: Serology (technique not specified)	No ^	Abortion rate was 0.6% in positive and 2.7% in negative cows. Incidence of stillbirth was 3.5% in positive and 1.9% in negative cows.	^*^^	Unclear
Turkey Kilicoglu et al., 2023 [64]	PR *	A: 670 aborted foetuses	OTS ^	DIR *: PCR on aborted calves’ livers	No ^	Total of 7.0% of samples were positive.	*^*^	Yes
United Arab Emirates Barigye et al., 2021 [65]	PR *	A: 759	CCS *	OTH ^: ELISA on serum	Yes *	Of all cows 36.5% were positive and 19.5% had previously aborted. Positive relationship between seropositivity and abortion history (*p* = 0.001).	**^*	Yes
United Kingdom Pritchard et al., 2011 [66]	PR *	A: 124	OTS ^	DIR *: Modified Ziehl-Neelsen stained impression smears of placental cotyledons; PCR of cotyledons and foetal fluids	No ^	*C. burnetii* found in 7.3% of samples.	*^*^	Yes
Uruguay Macias-Rioseco et al., 2019 [67]	PR *	A: 4 placentas/foetuses from *C. burnetii*-associated aborted cows H: 1	OTS ^	DIR *: Histology, IHC and PCR on placentas	No ^	Total of 2/4 placentas had evidence of fibrinonecrotizing placentitis. *C. burnetii* was found intralesionally by IHC.	*^*^	Yes
Uruguay Macias-Rioseco et al., 2020 [68]	PR *	A: 102 cows that aborted	OTS ^	DIR *: IHC on placentas and/or foetuses	No ^	Total of 6% of abortion cases were considered to be due to *C. burnetii*.	*^*^	Yes

A scientific evidence score was calculated for each study based on four major criteria to help reach a conclusion on the level of evidence for the impact of *C. burnetii*, if any, on the reproductive parameters assessed. Type of publication: peer-reviewed (PR *); other publication (OP ^). Type of study: case–control and cohort studies (CCS *); other type of study (without a control group; OTS ^). Type of *C. burnetii* test: direct test (PCR, histology, ELISA antigen) on individual samples (DIR *); indirect test (antibodies based on serology) or direct test on a sample of pooled animals (including Bulk Tank Milk—BTM) (OTH ^). Comparative statistical analysis: Yes *; No ^. If all four criteria are met (PR, CCS, DIR and Yes), a score of **** is assigned; for each criterium not met, a * is replaced by a ^. All evidence scores contain four characters, either * or ^.

The studies in Table 3 that have the highest evidence scores based on type of publication, inclusion of a control group, type of test for identification of *C. burnetii* and applied comparative statistical analysis, and that were published over the last 10 years (since 2014, the date of the last published review by Garcia-Ispierto and colleagues [5]) are discussed in more detail below. In addition, several of the studies in Table 3 provide useful information regarding the location of the bacteria using PCR and IHC, and some indications of the mechanism of action and life cycle of the bacteria as it relates to reproduction, including the ASPW complex.

Many studies that looked at individual cows found that seropositivity was associated with the prevalence of the ASPW complex. For example, a recent study [53] reported that the seropositivity rate of aborted cows was higher than that of non-aborting cows. Also, based on the presence of *C. burnetii* DNA in BTM and/or aborted foetuses, they noted that 18.3% of herds were positive. One stillbirth-specific study [45] looked at stillbirth and live birth calves born into an endemically *C. burnetii*-infected herd. This study showed that 7.1% of precolostral blood samples from the stillbirth calves were PCR-positive for *C. burnetii*, while none of the live birth calves were infected. In addition, *C. burnetii* DNA was found in 5/12 cotyledon samples collected transvaginally from cows with stillbirth calves, while it was detected in only 2/12 cows with live birth calves. These findings raise the question of potential bacteraemia or septicaemia explaining the death of the calves at the end of pregnancy. Mangena and colleagues [57] provide some additional information in a surveillance study in South Africa. Despite a low seropositivity rate (0.7% in 272 cows tested), *C. burnetii* DNA was found in organs of aborted foetuses and stillborn calves in 2/6 animal samples. Another recent controlled study performed in one large herd in the UAE [65] found that approximately 34% of seropositive cows versus 11% of seronegative cows had a history of abortion. The odds ratio of prior abortion was 4.3 times higher in seropositive than in seronegative cows.

Several studies measuring the incidence of *C. burnetii* in the field have also looked at other possible abortion-causing pathogens. For example, Derdour and colleagues [27] found that seropositive cows were seven times more at risk of abortion than seronegative cows. However, the authors considered that *Neospora caninum*, *Leptospira* Hardjo, Bovine Herpes Virus 4 (BoHV-4) and *Brucella abortus* posed a greater abortion risk than *C. burnetii* in Algeria. Similarly, in a study in India [48], *C. burnetii* DNA was detected in 12/64 samples from aborted cows. However, seropositivity and history of abortion were not associated, but of the 64 abortion cases investigated, antibodies to bovine viral diarrhoea virus (BVDV), *B. abortus*, bovine herpes virus 1 (BoHV1), *L.* Hardjo, *N. caninum* and *C. burnetii* were detected in 63, 61, 56, 35, 5 and 6 cows, respectively. A study [35] in Ethiopia found a 22.6% prevalence of abortion in cattle. Of these, *C. burnetii*-seropositive cows (8.7% of the total) were more likely to abort than seronegative cows, but the authors concluded that cattle abortion in Ethiopia has mostly been associated with *B. abortus*, *C. burnetii*, *L.* Hardjo and the coinfection of these three pathogens. A smaller study in Switzerland [60] found that *C. burnetii* is a major cause of infectious perinatal mortality, more than any of the other 13 pathogens tested for. Not all studies show a positive connection between ASPW complex and *C. burnetii* infection. For example, Valla and colleagues [51] found no relationship between *C. burnetii* positivity (based on BTM PCR) and the incidence of abortion at the herd level. However, in that study, the other possible causes of abortion were not investigated. Seropositivity data can sometimes raise questions regarding measurement methodology for *C. burnetii* infection and shedding. For example, a recent study [47] carried out on 44 herds with a history of abortion and other reproductive disorders found that 44% of blood samples were seropositive for *C. burnetii*, of which 60% were associated with abortions. All seropositive cows experienced reproductive troubles, either abortions or other reproductive disorders. However, the authors hypothesized that seropositive animals may not necessarily be currently infective as they can be PCR-negative, while seronegative ruminants can actively shed *C. burnetii*. Indeed, based on the reviewed literature, questions remain regarding how and when to best apply the diagnostic methodology and whether any impact of *C. burnetii* on the ASPW complex can be influenced by the stage of pregnancy. Thus, although a few studies have either been inconclusive or showed no significant relationship between *C. burnetii* infection and abortions, it seems quite clear that *C. burnetii* is one of several causal agents for the ASPW complex in cattle. While reviewing the literature that met the systemic review search criteria, it was noted that 9/43 studies provided some data that could shed light on possible mechanisms of action or pathogenesis related to *C. burnetii* and the ASPW complex. For example, using an initial modified-acid fast (MAF) smear test of fresh aborted placenta samples to identify *C. burnetii* infections, Bildfell and colleagues [31] then used IHC to determine the presence or absence of *C. burnetii* in 14 smear-positive and 29 smear-negative samples. Nine and one samples were IHC-positive in the smear-positive and smear-negative samples, respectively. IHC-positive samples, but not IHC-negative ones, were significantly associated with placental inflammation (*p* = 0.003), placental necrosis (*p* = 0.012), foetal pneumonia (*p* = 0.015) and the visibility of *Coxiella*-like organisms within trophoblasts using stained sections. The IHC-positive placentas also included infiltration of the chorionic stroma by mononuclear cells, necrosis of chorionic trophoblasts, and focal exudation of fibrin and neutrophils. Subsequently, a Danish study [26] compared a fluorescent in situ hybridization (FISH) assay with IHC for the detection of *C. burnetii* in samples of aborted placentas in cattle, where the abortions were determined to be due to *C. burnetii*. The bacterium was detected in all 12 samples by both methods, specifically within the cytoplasm of single trophoblast cells, within mononuclear cells and in the placental debris. Another Danish study [69], which did not fit within the publication search criteria, analysed (using PCR) cotyledon samples from 170 dairy cows (after normal calving) for the presence of *C. burnetii*. The animals chosen in this study originated from a mixture of *C. burnetii* BTM (ELISA)-positive (*n* = 9), -intermediate (*n* = 2) and -negative (*n* = 8) herds used in a previously published *C. burnetii* surveillance study [70]. Ninety PCR-positive and 20 PCR-negative cotyledon samples were used for histology and IHC. The authors noted that the main lesions observed in aborted cows were mild to moderate vasculitis, thrombosis or necrotizing placentitis. However, they concluded that *C. burnetii* infection in the placentas of parturient cows was rarely associated with inflammation, and the lack of lesions may explain why bovine Q fever is mostly subclinical. Furthermore, they concluded that cattle originating from herds with negative BTM antibody levels may shed *C. burnetii* at calving. 

Abortion materials from 124 aborting cows were examined in the UK [66] for the presence of *C. burnetii*. Using routine modified Ziehl–Neelsen (ZN) stained impression smears of placental cotyledons, the bacterium was not found, but nine of the samples (7.3%) were found to be positive based on PCR tests of the cotyledons. However, evidence for prevalence and association based on ZN staining should be treated with caution due to the lack of sensitivity [71] and possible misclassification associated with the ZN staining tool. In another study [54] with 100 aborted and/or stillbirth foetuses submitted for postmortem examination, samples of pooled foetal tissues (brain, heart, liver and lung) and placental cotyledons were tested for *C. burnetii* by PCR while the cotyledons were also tested using IHC. Four samples were positive based on IHC and all of these were also positive based on PCR; interestingly, all these were from third-trimester calves and were associated with inflammation of the placenta. Five other cotyledon samples that were IHC-negative were strongly positive based on PCR, even though their associated foetal organs were negative or weakly positive. A recent study in Uruguay [72] investigated a case of *C. burnetii* abortion in one dairy cow, which exhibited severe necrotizing placentitis with abundant intratrophoblastic and intralesional *C. burnetii*, as confirmed by IHC and PCR analyses. A seminal study [73] previously highlighted that Q fever-associated abortion in cattle is characterized by severe, acute, predominantly haemorrhagic and necrotic placentitis. An in vitro study [74] aimed to elucidate the interactions between *C. burnetii* and epithelial cells of the bovine host. Their findings suggested that epithelial cells, particularly mammary epithelial cells, may serve as a reservoir for *C. burnetii* replication in vivo, potentially evading the host’s immune response. Notably, *C. burnetii* exhibited greater proliferation within the foetal placenta compared with the maternal placenta, suggesting a potential link between foetal infection and abortion. In a separate study, Kilicoglu and co-workers [64] examined 670 aborted calf liver samples for *C. burnetii* via PCR, with 7% testing positive. Comparative analysis revealed elevated malondialdehyde and nitric oxide levels, alongside decreased glutathione levels in positive samples, indicating that abortions associated with foetal liver infection by *C. burnetii* may correlate with alterations in free radical levels and antioxidant activity. Garcia-Ispierto and colleagues [75] investigated the effects of *C. burnetii* seropositivity on hormonal patterns in blood among 58 pregnant non-aborting cows. Their results indicated that *C. burnetii* antibody levels influenced cortisol, progesterone and pregnancy-associated glycoproteins (PAG). Seropositivity was associated with placental damage and reduced PAG levels during the latter half of gestation, accompanied by elevated cortisol levels on day 180 of gestation, suggesting potential placental damage and abortion risk. These findings suggest that placentitis following *C. burnetii* infection may precipitate abortion, possibly through mechanisms involving decreased progesterone and PAG levels and direct foetal infection leading to abortion post-foetal demise.

### 3.3. Retained Placenta/Foetal Membranes (RFMs)

The bibliographic search yielded only nine publications addressing the association between *C. burnetii* and the incidence of RFMs (Table 1 and Table 4). Of these, three had evidence scores of three or four (Table 1), and two of these indicated an association (Table 2). If all four criteria are met (PR, CCS, DIR and Yes), a score of **** is assigned; if one criterion is not met: ***^; if two are not met: **^^; if three are not met: *^^^; if no criteria are met: the total evidence score cell shows ^^^^. All evidence scores contain four characters, either * or ^.

**Table 4:** Exhaustive list of studies (*n* = 9) related to the putative involvement of *C. burnetii* in RFMs.

Country Authors Ref. No.	Type of Publication: PR or OP	Study Population: no. of Animals (A) and/or Herds (H)	Type of Study: CCS or OTS	Type of Test to Identify Presence of *C. burnetii*: DIR and/or OTH	Comparative Statistical Analysis: Yes or No	Key Findings	Evidence Score (^^^^ to ****)	Evidence for Association between RFMs and *C. burnetii:* Yes, No or Unclear
Bulgaria Vidic et al., 1990 [29]	PR *	A: 165 with RFMs, 1.645 random cows	OTS ^	OTH ^: CFT on serum	Yes *	Total of 10.9% of cows had *C. burnetii* antibodies. There was no association with RFMs.	*^^*	No
Greece Dovolou et al., 2011 [46]	PR *	H: 80 (for global seroprevalence) A: 526; H: 10 (for within herd seroprevalence and relationship with fertility disorders)	OTS ^	OTH ^: ELISA on BTM for global seroprevalence; ELISA on blood for individual seroprevalence	Yes *	Total of 35% (*n* = 28) of the farms have antibodies in BTM. No clear statistical relationship between animal serology status and RFM rate.	*^^*	No
Hungary and Slovakia Dobos et al., 2020 [76]	PR *	A: 72 cows with RFMs; H: 15	OTS ^	DIR *: PCR and IHC on cotyledons	No ^	Positivity in approx. 61% of samples indicating a link to RFMs.	*^*^	Yes
Hungary Dobos and Fodor 2021 [77]	PR *	A: 167 monitored for RFMs; H: 35	CCS *	DIR *: PCR on cotyledons	Yes *	Total of 89% of 90 RFM cow cotyledons and 40.3% of 77 non-RFM cow cotyledons were PCR-positive (OR 12.6; *p* = 0.002). Total of 21.3% of RFM cow samples were highly loaded with *C. burnetii* vs. none of the non-RFM samples.	****	Yes
Iran Khalili et al., 2012 [78]	PR *	A: 161 (79 with reproductive problems); H: 19 (9 with reproductive problems)	CCS *	OTH ^: ELISA on serum	Yes *	Total of 51.4% of cows with reproductive problems (abortion, stillbirth, RFMs, mastitis) vs. 10.3% without problems were positive (*p* < 0.05).	**^*	Yes
Pakistan Rashid et al., 2019 [79]	PR *	A: 827 (cows and buffaloes); H: 11	OTS ^	OTH ^: ELISA on serum	Yes *	Seropositivity of 6.1%. Seropositive animals were more likely (OR 1.68; 95% CI 0.94–5.73; no *p*-value) to have RFMs.	*^^*	Yes
Spain Lopez-Gatius et al., 2012 [58]	PR *	A: 781 (> 90 day in milk); H: 3 (history of sub-fertility and positive BTM tests)	OTS ^	OTH ^: PCR on BTM; ELISA on serum	Yes *	Seropositive cows (50.2%) were more likely to have RFMs (OR 1.74; 95% CI 1.31–3.42; *p* = 0.04) than seronegative cows.	*^^*	Yes
Spain Garcia-Ispierto et al., 2013 [80]	PR *	A: 43; H: 2	CCS *	DIR * & OTH: PCR on BTM, individual milk, colostrum, faeces, cotyledons, vaginal fluid; ELISA on serum	Yes *	No effect of *C. burnetii* on incidence of RFMs.	****	No
Turkey Yilmazbas-Mecitoglu et al., 2022 [63]	OP ^	A: 575 (165–170 day pregnant)	CCS *	OTH ^: Serology (technique not specified)	No ^	Incidence of RFMs was 9.4% in positive (*n* = 170) and 14.5% in negative (*n* = 214) cows.	^*^^	No

A scientific evidence score was calculated for each study based on four major criteria to help reach a conclusion on the level of evidence for the impact of *C. burnetii*, if any, on the reproductive parameters assessed. Type of publication: peer-reviewed (PR *); other publication (OP ^). Type of study: case–control and cohort studies (CCS *); other type of study (without a control group; OTS ^). Type of *C. burnetii* test: direct test (PCR, histology, ELISA antigen) on individual samples (DIR *); indirect test (antibodies based on serology) or direct test on a sample of pooled animals (including Bulk Tank Milk—BTM) (OTH ^). Comparative statistical analysis: Yes *; No ^. If all four criteria are met (PR, CCS, DIR and Yes), a score of **** is assigned; for each criterium not met, a * is replaced by a ^. All evidence scores contain four characters, either * or ^.

Of the nine studies addressing RFMs, just over half found an association between *C. burnetii* infection and RFMs. For example, Spanish scientists [58] studied three high-producing dairy herds infected by *C. burnetii* that had a history of sub-fertility. Antibodies to *C. burnetii* were found in half of the cows. They found that seropositivity (50.2% of cows) increased the chances of an RFM by nearly a factor of two. Khalili and colleagues [78] in Iran tested cows for *C. burnetii* antibodies from herds with reproductive problems and herds without apparent problems. Problems examined included abortion, stillbirth, RFMs and mastitis. The data showed that 51.3% of cows from herds with problems and 10.3% of cows from herds without problems were positive for *C. burnetii*. In addition, all problem farms had at least one seropositive animal, while 80% of the “healthy” farms had only seronegative animals. Similarly, Rashid and colleagues [79] determined the presence of *C. burnetii* antibodies in the sera of cows and buffaloes. Across all animals, in comparison with “no disorder”, RFMs and ovarian disorders were significantly associated with seropositivity when a binary logistic regression model was used. When a univariate analysis was performed, seropositive animals were also 1.7 times more likely to have RFMs. The other parameters assessed (infertility, uterine disorders, premature birth/repeat breeder and history of abortion) were not significantly associated with seropositivity. A non-controlled study carried out in Hungary and Slovakia aimed to investigate the importance of *C. burnetii* in RFMs in dairy cows [76]. Cotyledons of placentas of cows that did not expel their placenta were tested for *C. burnetii* using both PCR and IHC tests which revealed positivity of 65.2% and 57.1%, respectively. The authors suggested that the high prevalence and shedding could be linked to RFMs; however, as the placenta is frequently contaminated by *C. burnetii*, even in cases of normal expulsion of the foetal membranes, conclusions should be drawn with caution. In a follow-up study by the same group [77], 88.9% of cotyledons from RFM cows and 40.3% of cotyledons from normally separated placenta cows tested positive for *C. burnetii* by PCR. Nearly a quarter of the positive samples from RFMs were highly loaded (>10^5^ CFU/mL) with *C. burnetii*, while the rest of the positive samples were moderately loaded (between 10^2^ and 10^5^ CFU/mL). They also found that RFMs were much more likely to be found in positive cows. They suggested that collecting and destroying placentas and aborted foetuses could be a useful safety measure employed to reduce the spreading of the bacterium to other animals and humans.

In contrast to the above, several studies found no association between *C. burnetii* and RFMs. For example, one of the earliest studies in this area [30] found that the seropositivity rate was not higher in cows having had RFMs than in randomly chosen cows. Similarly, in a small study involving 43 dairy cows, Garcia-Ispierto and colleagues [80] found that seropositive cows were no more likely to have an RFM than seronegative cows. Furthermore, a large study in Greece [46] grouped herds according to BTM antibody titre to *C. burnetii*. One-third of herds were deemed positive, and two herds per each of five herd groups (including a control group) were selected for further serum antibody titre assessments. The prevalence of seropositive animals between herds varied from 4.9 to 46.3%, and some positive animals were even found in the negative BTM herds. Various comparisons were performed (see also Table 5 and Table 6) but the authors felt that some apparent differences were not possible to interpret biologically. A more recent study [63] compared *C. burnetii* seropositive cows with seronegative cows. The incidence of RFMs was 14.5% in seronegative cows and 9.4% in seropositive cows, but no statistical analysis was presented.

The available evidence provides limited indications that *C. burnetii* infection could potentially have a direct or indirect correlation with RFMs. However, extensive, well-designed studies on a larger scale are imperative to conclusively elucidate and confirm this relationship.

### 3.4. Metritis and Endometritis

Only eight publications have examined the possible effects of *C. burnetii* on metritis and/or endometritis (Table 1 and Table 5). Of these, four had evidence scores of three or four (Table 1), and two indicated an association (Table 2).

**Table 5:** Exhaustive list of studies (*n* = 8) related to the putative involvement of *C. burnetii* in metritis and/or endometritis.

Country Authors Ref. No.	Type of Publication: PR or OP	Study Population: no. of Animals (A) and/or Herds (H)	Type of Study: CCS or OTS	Type of Test to Identify Presence of *C. burnetii*: DIR and/or OTH	Comparative Statistical Analysis: Yes or No	Key Findings	Evidence Score (^^^^ to ****)	Evidence for Association between (Endo) Metritis and *C. burnetii:* Yes, No or Unclear
Canada Turcotte et al., 2021 [81]	PR *	A: 202; H: 9	OTS ^	DIR * & OTH: PCR on vaginal mucus; ELISA on milk	Yes *	All vaginal samples were negative. Total of 12.9% of milk samples were positive. No link of positivity with cytological endometritis.	*^**	No
Greece Dovolou et al., 2011 [46]	PR *	H: 80 (for global seroprevalence) A: 526; H: 10 (for within herd seroprevalence and relationship with fertility disorders)	OTS ^	OTH ^: ELISA on BTM for global seroprevalence; ELISA on blood for individual seroprevalence	Yes *	Total of 35% (*n* = 28) of the farms have antibodies in BTM. No statistical relationship between animal serology status and uterine infection.	*^^*	No
Hungary Dobos et al., 2022 [82]	PR *	A: 40 infertile cows; H: 5	OTS ^	DIR * & OTH: PCR on uterine swabs; histology on uterus biopsies; ELISA on serum	Yes *	Total of 65% of cows were seropositive. *C. burnetii* DNA found in 7.5% of swabs/biopsies. Total of 41% of samples had moderate/severe cell infiltration of the endometrium.	*^**	Yes
Italy Valla et al., 2014 [51]	PR *	H: 246 (106 positive)	CCS *	OTH ^: PCR on BTM	Yes *	Positive herds more likely (OR 2.49; *p* = 0.0005) to have metritis/clinical endometritis.	**^*	Yes
Italy De Biase et al., 2018 [83]	PR *	A: 40	OTS ^	DIR *: IHC and PCR on uterine biopsies	No ^	Total of 25% of samples were PCR-positive and had mild/severe chronic endometritis.	*^*^	Yes
Netherlands Muskens et al., 2011 [84]	PR *	A: 45 cows with metritis; H: 12	OTS ^	DIR * & OTH: PCR on uterine discharge; ELISA on BTM	No ^	One uterine sample tested positive. Four cows were seropositive. No link to metritis.	*^*^	No
Spain Garcia-Ispierto et al., 2013 [80]	PR *	A: 43; H: 2	CCS *	DIR * & OTH: PCR on BTM, individual milk, colostrum, faeces, vaginal fluid; ELISA on serum	Yes *	Seropositive cows were less likely (OR 0.10) to suffer endometritis. Animals not infected with *C. burnetii* seem to be susceptible to infection and not protected against the bacterium.	****	Unclear
Turkey Yilmazbas-Mecitoglu et al., 2022 [63]	OP ^	A: 575 (165–170 day pregnant)	CCS *	OTH ^: Serology (technique not specified)	No ^	Incidence of metritis was 7.1% in positive (*n* = 174) and 8.4% in negative (*n* = 226) cows.	^**^	No

A scientific evidence score was calculated for each study based on four major criteria to help reach a conclusion on the level of evidence for the impact of *C. burnetii*, if any, on the reproductive parameters assessed. Type of publication: peer-reviewed (PR *); other publication (OP ^). Type of study: case–control and cohort studies (CCS *); other type of study (without a control group; OTS ^). Type of *C. burnetii* test: direct test (PCR, histology, ELISA antigen) on individual samples (DIR *); indirect test (antibodies based on serology) or direct test on a sample of pooled animals (including Bulk Tank Milk—BTM) (OTH ^). Comparative statistical analysis: Yes *; No ^. If all four criteria are met (PR, CCS, DIR and Yes), a score of **** is assigned; for each criterium not met, a * is replaced by a ^. All evidence scores contain four characters, either * or ^.

If all four criteria are met (PR, CCS, DIR and Yes), a score of **** is assigned; if one criterium is not met: ***^; if two are not met: **^^; if three are not met: *^^^; if no criteria are met: the total evidence score cell shows ^^^^. All evidence scores contain four characters, either * or ^. Some studies address the association of *C. burnetii* infection with uterine infections in general, while other studies focus either on metritis or endometritis. A study in Turkey [63] compared *C. burnetii* seropositive cows with seronegative cows. The incidence of metritis was 8.4% in seronegative cows and 7.1% in seropositive cows. Although no statistical analysis has been presented by the authors, one can assume that the difference is not significant. Again, the usefulness of serology to categorize active *C. burnetii*-infected cows is questionable, especially when looking at multi-factorial conditions like metritis and endometritis. A small study [82] with 40 infertile dairy cows found that 65% of animals were seropositive for *C. burnetii* while only 7.5% were PCR-positive for uterine swabs and biopsy samples. Although 41% of samples had moderate/severe cell infiltration of the endometrium, subsequent histological examination of the biopsies revealed no clear link between the severity of noted endometrial lesions and the pathogenicity of the bacteria. The authors suggested that serological data and PCR detection of the pathogen in the uterus may not be correlated. However, it is important to note that there were no fertile control cows in the study, so the conclusions must be treated with caution. In another more detailed study [84], Muskens and colleagues tested the uterine content of 45 dairy cows with puerperal metritis for *C. burnetii* using PCR but found that only one sample tested positive even though four cows had antibodies to *C. burnetii*. The authors concluded that, in this case, *C. burnetii* was not a major aetiology of puerperal metritis. A study in Greece [46] also found no association between the presence of *C. burnetii* antibodies and uterine infection. Turcotte and colleagues [81] conducted a study about 5 weeks after calving to estimate the prevalence of *C. burnetii* in dairy cows from PCR-positive and/or seropositive herds. All 202 PCR-assayed vaginal samples were negative for *C. burnetii*. However, 13% of the milk samples were seropositive. No primiparous cows were seropositive, while 17–20% of second- and third-parity cows were seropositive. The relationships of reproductive disorders (using purulent vaginal discharge, cytological endometritis and success at first service as indicators) to seropositivity were not significant, but the number of animals was too low to fully examine these parameters. Furthermore, no other analysis targeting other pathogens responsible for metritis was carried out. It is therefore difficult to draw a definitive conclusion. The authors also noted that the incidence of seropositivity would likely have been higher if the milk samples were collected closer to parturition and that the impact of the bacterial infection could depend “on the state of the infection, acute or chronic, and could vary according to the animal immunity”. The absence of detected shedding may also imply that the bacterium was not actively circulating in the herd at the time of the study. 

In contrast to the above, Valla and colleagues [51] found that about 40% of 344 dairy herds were PCR-positive for *C. burnetii* in BTM. Data on the incidence of metritis and endometritis were recorded on 246 farms. A significant incidence of metritis/endometritis in a herd was defined as >15–17%, and this was seen in 43.1% of the farms. Based on this definition, positive herds were more likely to have metritis/clinical endometritis than negative herds. Because the abortion rate was not significantly different between positive and negative herds, the increased incidence of metritis does not seem to be related to abortion. However, in this study, a direct aetiological diagnosis of clinical metritis/endometritis was not made. Consequently, the direct responsibility of *C. burnetii* in these uterine infections could not be confirmed. Some studies have focused more specifically on endometritis. For example, to specifically examine the association between *C. burnetii* and chronic endometritis, De Biase and colleagues [83] performed IHC and PCR on uterine biopsies from infertile dairy cows; the biopsies were performed about 8 mo postpartum. A quarter of the animals were PCR-positive for *C. burnetii* and negative for other pathogens—these samples revealed mild to severe endometritis, and intralesional and intracytoplasmic *C. burnetii* were found in macrophages in the endometrium. Importantly, no significant histopathological changes were observed in *C. burnetii*-negative biopsy samples. The authors claimed that this was the first report describing the presence of *C. burnetii* in association with endometritis, uterine vasculitis and fibrosis. However, it is important to note that no fertile cows were included as controls. Another very recent study [85], which would have fit within the bibliographic research criteria if the search had been performed more recently, published in this special issue on Q fever in ruminants, used the presence of *C. burnetii* antibodies in BTM (*n* = 262 farms; *n* = 12,052 cows) to examine the relationship between *C. burnetii* infection and reproductive performance. One hundred and fifty-eight (60.1%) farms tested positive. In this study, puerperal metritis (acute) and endometritis (chronic) were assessed independently. According to the authors, positive farms had a higher incidence of endometritis than did negative farms (13.7% vs. 11.2%; *p* < 0.05). However, there was no effect of seropositivity on metritis (11.5% vs. 10.2% in seronegative cows). Some studies associate *C. burnetii*-infected animals with increased reproductive disorders; however, this is not always the case, and the discrepancy between studies may well be due to the types (serology vs. PCR vs. IHC) and timing of tests used, and what biological materials are tested. For example, a study in Spain [80] demonstrated that dairy cows not infected with *C. burnetii* seem to be susceptible to infection and are not protected against the bacterium. They found that the likelihood of suffering endometritis was 10 times lower in seropositive animals compared with seronegative animals. The number of animals in this study was not very large (and only one animal was seropositive), but the data do substantiate the theory that a seronegative result does not mean an animal is not infected [86].

Although some studies did not involve a control group, there is histological evidence that *C. burnetii* may be directly or indirectly associated with endometritis and/or metritis [83]. In addition, metritis and endometritis should be separated in future studies as *C. burnetii* may be more involved in endometritis than in puerperal metritis. Clearly there is a need for more, larger and better-designed studies to provide more evidence for or against the association of *C. burnetii* and endometritis/puerperal metritis. 

### 3.5. Infertility and Sub-Fertility

Only 19 studies have examined the possible effects of *C. burnetii* on infertility/sub-fertility (Table 1 and Table 6). Of these, 12 had evidence scores of three or four (Table 1), and 8 of these indicated an association (Table 2).

**Table 6:** Exhaustive list of studies (*n* = 19) related to the putative involvement of *C. burnetii* in infertility/sub-fertility.

Country Authors Ref. No.	Type of Publication: PR or OP	Study Population: no. of Animals (A) and/or Herds (H)	Type of Study: CCS or OTS	Type of Test to Identify Presence of *C. burnetii*: DIR and/or OTH	Comparative Statistical Analysis: Yes or No	Key Findings	Evidence Score (^^^^ to ****)	Evidence for Association between Reproductive Disorders and *C. burnetii:* Yes, No or Unclear
Belgium Saegerman et al., 2015 [29]	PR *	H: 206	OTS ^	OTH ^: ELISA on BTM	Yes *	BTM seropositive herds (58%) are more likely to experience irregular repeat breeding (OR 2.02; 95% CI 1.07–3.81; *p* = 0.03).	*^^*	Yes
Canada Turcotte et al., 2021 [81]	PR *	A: 202; H: 9	OTS ^	DIR * & OTH: PCR on vaginal samples; ELISA on individual milk	Yes *	Positivity and success at first service were not associated (OR 1.3; 0.48–3.3; *p* = 0.64).	*^**	No
Czech Republic Literak and Rodriguez 1994 [33]	PR *	A: 213	OTS ^	OTH ^: CFT on serum	Yes *	Total of 6.1% of aborting cows were seropositive. No association of seropositivity with other reproductive parameters.	*^^*	No
France Jegou et al., 2022 [40]	OP ^	H: 642	OTS ^	DIR * & OTH: PCR on vaginal discharge; ELISA on serum of six randomly chosen cows within the same herd	Yes *	First service fertility rate in herds with *C. burnetii*-associated abortions were 7 percentage points lower than in herds without *C. burnetii*-associated abortions (40.2% vs. 47.2%; *p* = 0.02).	^^**	Yes
Germany Sting et al., 2000 [42]	PR *	A: 826; H: 38	OTS ^	DIR * & OTH: Capture ELISA on vaginal swabs (antigen detection); CFT on serum	Yes *	Higher *C. burnetii* antibody levels linked to greater insemination ratios (*p* > 0.05). Non-pregnant cows excreted more *C. burnetii* than pregnant cows (*p* < 0.05).	*^**	Yes
Germany Sting et al., 2002 [43]	PR *	A: 1167 (149 unsuccessfully inseminated); H: 105	CCS *	DIR * & OTH: Capture ELISA on vaginal swabs (antigen detection); ELISA on serum	Yes *	*C. burnetii* infections (antigen detected) are not associated with repeated inseminations without success (OR 1.16; 95% CI 0.75–1.78; *p* = 0.48). No association with seropositivity.	****	No
Germany Freick et al., 2017 [87]	PR *	A: 69; H: 1	OTS ^	DIR * & OTH: PCR on vaginal swabs and milk; ELISA on serum	Yes *	Vaginal *C. burnetii* shedding was highest at parturition (30.9%). *C. burnetii* seropositivity and shedding had no impact on any parameters (*n* = 7) of reproduction.	*^**	No
Greece Dovolou et al., 2011 [46]	PR *	H: 80 (for global seroprevalence) A: 526; H: 10 (for within herd seroprevalence and relationship with fertility disorders)	OTS ^	OTH ^: ELISA on BTM for global seroprevalence; ELISA on blood for individual seroprevalence	Yes *	Total of 35% (*n* = 28) of the farms have antibodies in BTM. No statistical relationship between animal serology status and fertility parameters [number of artificial inseminations (AIs) per pregnancy, calving-to-calving interval].	*^^*	No
Hungary Dobos et al., 2020 [88]	PR *	A: 321 cows pregnant 29–35 day after AI; H: 3	CCS *	OTH ^: ELISA, CFT on serum	Yes *	Total of 52% of cows were ELISA-positive. Positive correlation (*p* < 0.05) between seropositivity and loss of pregnancy (60–70 day after AI). High rate (18%) of pregnancy loss.	**^*	Yes
India Dhaka et al., 2020 [89]	PR *	A: 711 (including 168 buffalo); H: 8	OTS ^	DIR * & OTH: PCR on blood; ELISA on serum	Yes *	Seropositivity was 17.7% in cattle. Seropositivity (46 of 102 cows) was positively linked to reproductive disorders (OR 2.54; 95% CI 1.67–3.85; *p* = 0.00001) vs. 85 of 478 seronegative cows.	*^**	Yes
India Balamurugan et al., 2021 [47]	PR *	A: 323; H: 44 (with history of reproductive disorders)	OTS ^	DIR * & OTH: PCR and ELISA on serum	Yes *	Overall seropositivity was 44% of which 35% were associated (*p* < 0.01) with (undefined) reproductive disorders.	*^**	Yes
Iran Khalili et al., 2012 [78]	PR *	A: 161; H: 19 (9 with and 10 without reproductive disorders)	CCS *	OTH ^: ELISA on serum	Yes *	Total of 51.35% with reproductive problems (such as abortion, stillbirth, RFMs, mastitis) and 10.3% without problems were seropositive (*p* < 0.05).	**^*	Yes
Italy Miotto et al., 2016 [90]	PR *	H: 28 (16 tested positive)	CCS *	OTH ^: PCR on BTM	Yes *	Number of available heats and days open were higher in positive herds (*p* < 0.05).	**^*	Yes
Pakistan Rashid et al., 2019 [79]	PR *	A: 827	OTS ^	OTH ^: ELISA on serum	Yes *	Cows with ovarian disorders (OR 1.79; 95% CI 0.22–14.37), infertility (OR 3.59; 95% CI 0.41–31.46), premature birth/repeat breeders (OR 8.98; 95% CI 2.17–37.21) were more likely to test seropositive.	*^^*	Yes
Spain Lopez-Gatius et al., 2012 [58]	PR *	A: 781 (>90 day in milk); H: 3 (history of sub-fertility and positive BTM tests)	CCS *	OTH ^: PCR on BTM; ELISA on serum	Yes *	Seropositive cows (50.2%) were less likely to have pregnancy loss (OR 0.57; 95% CI 0.33–0.99; *p* = 0.04) than seronegative cows. There was no clarity on the effect of seropositivity on parturition to conception interval.	**^*	Unclear
Spain Garcia-Ispierto et al., 2013 [80]	PR *	A: 43; H: 2	CCS *	DIR * & OTH: PCR on BTM, individual milk, colostrum, faeces, vaginal fluid; ELISA on serum	Yes *	Seronegative cows had delayed return to luteal activity (hazard ratio 2.55, 95% CI 1.4–3.4; *p* = 0.02. Conception was delayed in non-shedding cows (hazard ratio 2.3, 95% CI 1.0–3.6; *p* = 0.05).	****	Yes
Spain Lopez-Helguera et al., 2013 [91]	PR *	A: 359 controls, 360 vaccinated against *C. burnetii*; H: 1	OTS ^	OTH ^: ELISA on serum to pre-screen animals	Yes *	Vaccinated cows were 1.22 times more likely to conceive during the first 150 day in milk than control cows. In seronegative cows, the likelihood of pregnancy was 1.25 times higher in vaccinated cows. No effect of vaccination on subsequent fertility in seropositive cows.	*^^*	Yes
Spain Garcia-Ispierto et al., 2015 [92]	PR *	A: 208 controls, 212 vaccinated against *C. burnetii* after 1st parturition; H: 1	CCS *	OTH ^: ELISA on serum to pre-screen animals	Yes *	First parturition: control cows were more likely to have early foetal loss (OR 1.42; 95% CI 1.1–2.8; *p* = 0.04) than vaccinated cows.	**^*	Yes
Turkey Yilmazbas-Mecitoglu et al., 2022 [63]	OP ^	A: 575 (165–170 day pregnant)	CCS *	OTH ^: Serology (technique not specified)	No ^	First conception rate was 34.4% in positive (*n* = 151) and 29.8% in negative (*n* = 181) cows. Embryonic loss rate was 11.1% in positive (*n* = 99) and 6% in negative (*n* = 83) cows. Rate of repeat breeders was 4.6% in positive (*n* = 152) and 4.9% in negative (*n* = 181) cows.	^*^^	No

A scientific evidence score was calculated for each study based on four major criteria to help reach a conclusion on the level of evidence for the impact of *C. burnetii*, if any, on the reproductive disorders assessed. Type of publication: peer-reviewed (PR *); other publication (OP ^). Type of study: case–control and cohort studies (CCS *); other type of study (without a control group; OTS ^). Type of *C. burnetii* test: direct test (PCR, histology, ELISA antigen) on individual samples (DIR *); indirect test (antibodies based on serology) or direct test on a sample of pooled animals (including Bulk Tank Milk—BTM) (OTH ^). Comparative statistical analysis: Yes *; No ^. If all four criteria are met (PR, CCS, DIR and Yes), a score of **** is assigned; for each criterium not met, a * is replaced by a ^. All evidence scores contain four characters, either * or ^.

If all four criteria are met (PR, CCS, DIR and Yes), a score of **** is assigned; if one criterium is not met: ***^; if two are not met: **^^; if three are not met: *^^^; if no criteria are met: the total evidence score cell shows ^^^^. All evidence scores contain four characters, either * or ^. The effect of pathogens or treatments on parameters of fertility and sub-fertility are often difficult to study due to the many confounding effects and the large numbers of animals required to find statistically significant differences. It is no wonder, then, that the evidence for or against a link between *C. burnetii* infection and fertility/sub-fertility is currently unclear. However, 63% of the studies in this systemic review, including 67% of the publications with evidence scores of three and four, indicated an association (Table 2). 

An early study in 1991–1992 [33] found no link between *C. burnetii* infection and pregnancy rate. Regarding the prevalence of *C. burnetii* and *Chlamydia*, Sting and colleagues [42] examined the effect of herd fertility status. The seropositivity rate was higher in animals needing two or more AIs than in animals needing only one AI to get pregnant. In contrast, a follow-up study by the same group [43] demonstrated that neither *C. burnetii* infections (antigen) nor seropositivity were significantly associated with the number of inseminations, despite both *C. burnetii* antigen and antibody levels being significantly associated with an increased incidence of abortion. Dovolou and colleagues [46] looked at 80 dairy herds for the seroprevalence of *C. burnetii*. BTM samples were assayed by ELISA, and 35% of herds were considered positive—these were then graded as 1, 2, 3 and 4 based on antibody titre (ascending order). Sera from cows within two herds per grade were tested by ELISA, and the prevalence of seropositive cows ranged from 4.9 to 46.3%. The negative and four grades of positive herds were compared for the numbers of AIs per pregnancy, calving interval, uterine infection rate, extended oestrous cycle length rate and RFM rate, but no significant differences were found. However, as the numbers of herds and cows per grade were low, these data must be viewed cautiously. A Spanish group [58] conducted a study on three high-producing dairy herds with confirmed *C. burnetii* DNA in BTM and a history of sub-fertility. Antibodies to *C. burnetii* in blood were detected in half the cows. Seropositivity (50% of cows) was correlated with RFMs and changes in the interval from parturition to conception, with cows exhibiting lower intervals between parturition and conception having lower levels of seropositivity. Seropositivity was also linked to early pregnancy (cows conceiving before day 90 postpartum) and the successful maintenance of pregnancy during the early foetal period. Indeed, seropositive cows were only half as likely to have pregnancy loss than seronegative cows. In attempting to elucidate these latter findings, the authors proposed a hypothesis. They suggested that if cows conceiving early are at an increased risk of having a longer lifespan in the herd, and a longer lifespan in the herd heightens the risk of exposure to *C. burnetii*, then the positive association between early pregnancy and seropositivity might be a reflection of survival. Consequently, effective management practices related to high-milk-producing cows could potentially enhance the cows’ response to *C. burnetii* infection. Unlike studies on other reproductive parameters, some studies have assessed the impact of *C. burnetii* on infertility/sub-fertility by comparing non-protected animals with animals protected from infection by vaccination. So, the same research team extended their investigation into the association between *C. burnetii* infection and reproduction by initiating a study [91] focused on the impact of preventing *C. burnetii* infection through a vaccination approach. In a dairy herd known to be infected with *C. burnetii*, dairy cows were randomly assigned to control and vaccinated groups. The time to conception interval was available for over 600 cows. The results revealed that the vaccinated group had a 1.22-fold higher likelihood of conceiving within the first 150 day of lactation compared with the control group. When considering only seronegative cows in both groups, vaccination increased the likelihood of pregnancy by a factor of 1.25. Conversely, in seropositive cows, vaccination did not show a significant effect on fertility. This group [80] also reported that, in infected herds, *C. burnetii*-positive dairy cows were more likely to exhibit an earlier return to luteal activity and conceive earlier compared with non-infected cows. Despite the limited sample size (*n* = 43), the authors put forth a hypothesis to explain this perhaps unexpected finding. They suggested that seronegative cows might be more susceptible to infection and lack protection against *C. burnetii*, potentially contributing to the observed reproductive outcomes. Another follow-up study by the same group looked at the effect of vaccination against *C. burnetii* on various reproductive parameters in cows over two calving periods in a herd with endemic infection [92]. Cows were divided into controls and vaccinated (over both pregnancies) and balanced for seropositive (about 28% of both groups) and seronegative cows. The likelihood of early foetal loss was higher in controls than in vaccinated cows; this effect was even more pronounced in seronegative cows. Also, vaccinated seronegative cows were only half as likely to be sub-fertile (>3 AIs) compared with seronegative control cows. Combined, the data from this Spanish group can be interpreted as showing a relationship between *C. burnetii* infection and reduced reproductive performance. 

Survey data gathered from 206 herds in Belgium indicated that BTM seropositive herds were twice as likely to experience repeat breeding than seronegative herds [29]. Similarly, Miotto and colleagues [90] enrolled herds that either had or did not have a *C. burnetii* infection based on BTM sample testing by PCR. Subsequently, data were collected using a questionnaire approach. The data showed that the calving to conception interval was 56 day longer in infected herds than in control herds. In contrast, Freick and colleagues [87] examined the association between *C. burnetii* seropositivity or vaginal bacterial shedding and various reproductive parameters by following heifers from 9 mo of age until 150 day post-calving within one endemically infected herd. Up until calving, all heifers were seronegative, but 31% of animals had vaginal shedding at calving, and 15.3% had positive milk samples 100 day post-calving. Neither seropositivity nor shedding had any effect on the reproductive parameters measured until 150 day in milk. A study with 11 farms in Pakistan [79] determined the presence of *C. burnetii* antibodies in the sera of cows and buffalo. Using univariate analysis, across all animals, in comparison with “no disorder”, ovarian disorders, infertility and premature birth/repeat breeders were disorders significantly associated with seropositivity. Using a binary logistic regression model, in comparison with “no disorder”, ovarian disorders were significantly associated with seropositivity, while infertility and premature birth/repeat breeders were not. Likewise, Dobos and colleagues [88] found that half the dairy cows tested across three farms were seropositive for *C. burnetii*. Pregnancy loss was detected in 18% of cows up to day 70 of gestation, and these cows had a seropositivity rate of 80.5%, while the seropositivity rate was only 48.4% in cows without pregnancy losses. A study in India [89] found that *C. burnetii* was found in a quarter of cows. When the analyses also included buffaloes, 17.6% of animals without reproductive disorders were positive for *C. burnetii*, while there were twice as many (35.1%) positive in the group with reproductive disorders. However, the authors do not give a definition of “reproductive disorders”, which makes the data difficult to interpret. As mentioned in an earlier section, a Canadian study [81] demonstrated that the relationship between reproductive disorders and seropositivity was not significant when using indicators such as purulent vaginal discharge, cytological endometritis and success at first service, but the number of animals was too low to fully examine these parameters. Another recent study [47] examined herds with a history of reproductive disorders; one-third of cows with reproductive disorders had antibodies against *C. burnetii*. However, no control group was included in this study. Consequently, these findings should be considered with caution. The authors of a large study in France [40] concluded that the first service conception rate in herds with *C. burnetii*-associated abortions was 7 percentage points lower than in herds without *C. burnetii*-associated abortions. Likewise, success at first insemination was two times lower in infected herds. Similarly, Yilmazbas-Mecitoglu and colleagues [63] compared *C. burnetii* seropositive cows with seronegative cows for a variety of reproductive parameters. The first service conception rates were 29.8% and 34.4%, the numbers of inseminations per pregnancy were 1.56 and 1.44, the embryonic loss rates were 6.0% and 11.1% and the rates of repeat breeder cows were 4.9% and 4.6%, respectively. However, as the authors did not present any statistical analysis, it is impossible to assess if there is a significant relationship between *C. burnetii* infection and the reproductive parameters evaluated. A very recent study [93], which would have fit within the bibliographic research criteria if the search had been performed more recently, published in this special issue on Q fever in ruminants, aimed to evaluate animal reproductive capacity and productivity after abortion that was or was not related to *C. burnetii* for dairy cows and heifers that were either *C. burnetii*-positive (*n* = 148) or -negative (*n* = 149). Seropositivity was based on an ELISA using serum. There was no effect of seropositivity on subsequent pregnancy after the first AI, AI rate/pregnancy, prolonged cycles above 23 and 48 day or days open. In contrast, another recent study [85] used the presence of *C. burnetii* antibodies in BTM (*n* = 262 farms) to examine the relationship between *C. burnetii* infection and reproductive performance. Over 60% of the farms tested positive. According to the authors, the overall conception rate was lower on seropositive farms than on seronegative farms (37.1% ± 10.3 vs. 39.8% ± 11.7; *p* < 0.05). The authors found similar data for the first service conception rate (32.9% ± 11.8 vs. 36.1% ± 13.2; *p* < 0.05). However, no significant differences were observed for calving to the first AI interval and number of days open. 

Overall, the evidence seems to indicate a relationship between *C. burnetii* infection and adverse reproductive performance. However, to determine the extent of the effect *C. burnetii* has on fertility/sub-fertility, more controlled studies with larger numbers of animals need to be performed, and determining what merits a “*C. burnetii* infection” definition needs to be examined more closely. 

## 4. Conclusions

This literature review offers a comprehensive evaluation of existing data on the impact of Q fever on cattle reproduction. Most studies, especially those with evidence scores of three and four, suggest the involvement of *C. burnetii* infection in various reproductive parameters, including the ASPW complex, RFMs and infertility/sub-fertility. Particularly compelling evidence emerges regarding the ASPW complex, with 43 studies addressing this aspect. However, substantial gaps persist in research, notably concerning RFMs, infertility/sub-fertility and metritis/endometritis (considering metritis and endometritis separately), warranting larger animal cohorts in more controlled settings to provide clarity. As we endeavour to deepen our understanding of how *C. burnetii* infection in cows affects reproduction, the intricate interplay of direct and indirect effects, compounded by co-infections with other pathogens, management practices and environmental factors, underscores the complexity of this research domain.

One key recommendation arising from this review is to encourage authors to adopt more precise definitions and robust methodologies for detecting the presence of *C. burnetii*, as well as to strive for consistency in defining various reproductive parameters. Such improvements would likely increase the capacity of future reviews to offer stronger evidence regarding *C. burnetii* involvement and impact on studied parameters. Furthermore, adherence to the World Organization for Animal Health guidelines regarding the use of robust diagnostic tools is paramount for confirming *C. burnetii* involvement in reproductive parameters. For instance, in cases of abortions and stillbirths, samples from the placenta, vaginal discharges and aborted foetus tissues (spleen, liver, lung or stomach content) should ideally undergo diagnosis via IHC, in situ hybridization or PCR. While serological tests detecting specific IgG antibodies provide evidence of recent *C. burnetii* infection or past exposure and are sometimes preferred for practical reasons, they are not as definitive as PCR for case confirmation.

The observed reproductive effects not only impact farm management and economics but also contribute to environmental contamination. Given the zoonotic nature of Q fever, increased shedding of *C. burnetii* elevates the risk of human exposure and subsequent contraction of the disease. Therefore, proactive measures are essential to safeguard both animal and human health.

## Data Availability

Data is contained within the article.
